# How Did COVID-19 Affect Suicidality? Data from a Multicentric Study in Lombardy

**DOI:** 10.3390/jcm10112410

**Published:** 2021-05-29

**Authors:** Camilla Gesi, Federico Grasso, Filippo Dragogna, Marco Vercesi, Silvia Paletta, Pierluigi Politi, Claudio Mencacci, Giancarlo Cerveri

**Affiliations:** 1 Department of Mental Health and Addiction, ASST Fatebenefratelli-Sacco, 20157 Milan, Italy; gesi.camilla@asst-fbf-sacco.it (C.G.); filippo.dragogna@asst-fbf-sacco.it (F.D.); claudio.mencacci@gmail.com (C.M.); 2 Department of Mental Health and Addiction, ASST Lodi, 26900 Lodi, Italy; marco.vercesi@asst-lodi.it (M.V.); silvia.paletta@gmail.com (S.P.); giancarlo.cerveri@asst-lodi.it (G.C.); 3 Department of Brain and Behavioral Sciences, University of Pavia, 27100 Pavia, Italy; pierluigi.politi@unipv.it

**Keywords:** COVID-19, SARS-CoV-2, suicidal behavior, first emergency care, multicentric, Lombardy

## Abstract

The aim of the study was to describe the characteristics of subjects accessing the emergency rooms for suicidal behavior during the first epidemic wave of COVID-19 in three Emergency Departments (EDs) in Lombardy (Italy). A retrospective chart review was conducted for the period 8 March–3 June 2020, and during the same time frame in 2019. For all subjects accessing for suicidality, socio-demographic and clinical data were collected and compared between the two years. The proportion of subjects accessing for suicidality was significantly higher in 2020 than in 2019 (13.0 vs. 17.2%, *p* = 0.03). No differences between the two years were found for sex, triage priority level, history of substance abuse, factor triggering suicidality and discharge diagnosis. During 2020 a greater proportion of subjects did not show any mental disorders and were psychotropic drug-free. Women were more likely than men to receive inpatient psychiatric treatment, while men were more likely to be discharged with a diagnosis of acute alcohol/drug intoxication. Our study provides hints for managing suicidal behaviors during the still ongoing emergency and may be primary ground for further studies on suicidality in the course of or after massive infectious outbreaks.

## 1. Introduction

About 800,000 people worldwide die every year due to suicide and an even greater number attempts suicide or engages in self-injuring behaviors [1]. Up to 90% of suicides around the world are associated with mental disorders and substance abuse, including harmful use of alcohol [2]. However, a broad variety of environmental factors also contribute to suicidal behavior, many of which originate from the concurrent cultural, social and economic context [1]. Suicidal spectrum behaviors include a broad variety of manifestations, from suicidal thoughts and plans, to suicidal self-injuring and suicide attempts, to completed suicide [3,4]. Despite the fact that most subjects with suicidal thoughts do not attempt suicide, suicidal ideation may often precede suicide attempts. However, according to the ideation-to-action framework, the development of suicidal ideation and the progression from ideation to suicide attempts are distinct phenomena with distinct explanations and predictors [5].

Individuals with suicidality often are referred to Emergency Departments (EDs), and EDs also frequently provide care for people with other risk factors for suicide, such as serious mental illness, substance use, and chronic pain. Every month, the number of visits to EDs prompted by suicidality is considerable, accounting for about 4% of accesses yearly in the US [6,7]. In addition, suicidal behaviors may not only represent the overt reason for the access, but also emerge as part of a broader constellation of psychiatric symptoms or be hidden by other complaints, so that the ED consultation itself may end up unraveling a current suicide risk [8,9]. Therefore, the emergency room of the EDs is an especially privileged observatory for the whole spectrum of suicidal behaviors [10].

The effect of natural and man-made disasters on suicidality has been evaluated in previous studies. Despite some inconsistent report, most data indicate a significant impact, either immediate or delayed, of disasters on suicide behaviors [11,12,13,14]. This is likely due to the detrimental effect of collective emergencies on mental health and psychosocial well-being, as well as to the socio-economic upheaval brought about by a range of consequences of disasters, such as the death or injury of family members, the loss of employment and properties, and the disruption of community cohesion and support [15,16].

Data focusing on the effect of massive infectious outbreaks on suicide behaviors are sparse, consistently with the relatively rare occurrence of epidemics in the last decades. Only poor evidence is available about the Spanish Flu, infecting 500 million people between 1918 and 1919 and narratively associated with a high risk of enacting suicidal behaviors among survivors [17]. During the more recent outbreak of SARS in Honk-Hong in 2003, rates of suicide were shown to rise compared to the previous year among elderly females, but not among elderly males or younger age groups. A recent nationwide cohort study conducted in Taiwan found significant higher rates of suicide, anxiety, depression, sleep- and trauma-related disorders among SARS survivors compared to non-affected subjects in the five years following the 2003 outbreak [18]. As for referral to EDs for suicidality, a study evaluating accesses to the emergency room in a SARS-dedicated hospital in northern Taiwan during 2003 SARS outbreak found an increased number of suicide attempts from drug overdoses during peak- versus pre-epidemic stages, despite the difference not being statistically significant [19].

The ongoing pandemic due to Sars-Cov-2 has obvious similarities with previous outbreaks, but also bears a few differences. After beginning in China in 2019, the COVID-19 has rapidly spread on a global scale with multiple epidemic waves in 2020. At the time of writing this paper, about 100 million people [20] have contracted the virus globally and more than two million have died. Besides the massive toll in terms of mortality, the health-related and social costs of COVID-19 are thought to be as much as significant. The impact on mental health is expected to be especially severe as the coronavirus epidemic has shown to enhance several relevant risk factors for mental illness, spanning from the loss of community life to widespread poverty, from unemployment to disruption of critical mental health and social services. Noteworthily, the compulsory quarantine enforced for preventing the propagation of the virus led to a sharp increase in social isolation and to a significant decrease in social support, which are among the most important risk factors for any kind of suicidal behavior [21,22]. Conversely, although data on deaths by suicide during the lockdown are still scarce, the first months of the pandemic might have been characterized by a lower suicide mortality rate [23]. For instance, a decrease of suicidal behaviors was observed in France during the strict lockdown. This decrease may be explained by several factors: the so-called “pulling-together effect”, observed in times of national tragedies, the work adaptation (reduced working hours and work-from-home policies), the subsidies limiting financial distress, the reduced access to illegal drugs. However, the absolute number of violent or severe suicide attempts remained relatively stable [24].

Italy was the first western country struck from the coronavirus pandemic. The first hotbed of contagion emerged at the end of February 2020 in Codogno, in the province of Lodi, about forty kilometers southeast of Milan, leading quickly to a quarantine setting enforced by law and to the rapid spread of fear. Besides the closure of schools, bars, restaurants and shops, the ED of Codogno was also temporarily closed to new admissions, and most patients were diverted to the neighboring hospitals of Pavia and Lodi. At the beginning of March, as the coronavirus reached the metropolitan area of Milan and started circulating across northern Italy, the entire Lombardy was placed on lockdown.

The main objective of the study was to describe the sociodemographic and clinical features of subjects accessing the psychiatric emergency service for suicidality during the first Sars-CoV-2 epidemic wave in three EDs in Lombardy, and to compare rates and characteristics of accesses between 8 March and 3 June 2020 to those occurring during the same period in 2019. We included accesses prompted by the whole spectrum of suicidal behaviors [4] (i.e., suicidal thoughts, suicidal self-injuring, suicide attempts, completed suicide) hereafter referred to as “suicidality” throughout the manuscript. The three EDs were chosen as differently hit by the epidemic, according to their distance from the first epicenter of the outbreak. In particular, Lodi-Codogno was the first center struck by Sars-CoV-2 epidemic in Italy and very severe restrictions were soon enforced in the attempt to prevent further spreading of the contagion. Pavia was involved in a second time in the epidemic wave, while the overflow of patients from Codogno was diverted to its hospital. Only in a later time the Sars-CoV-2 wave reached the metropolitan area of Milan, as the epidemic was already spreading across the whole of Lombardy.

## 2. Materials and Methods

### 2.1. Materials and Methods

A retrospective observational study was conducted at three EDs (Lodi-Codogno, San Matteo-Pavia, Fatebenefratelli-Milan) in Lombardy. The ED of Lodi-Codogno, where the first indigenous case of COVID-19 in Italy was confirmed, comprises two emergency rooms located in southern Lombardy, with a catchment area of about 230,000 inhabitants. The ED of San Matteo Hospital in Pavia, located 38 Km west of Lodi-Codogno and usually covering a district of about 550,000 inhabitants, during the first outbreak served to handle the overflow from the neighboring hospitals of Codogno and Lodi, which rapidly became overwhelmed. The ED of Fatebenefratelli Hospital is located in the metropolitan area of Milan (30 and 40 Km north of Lodi and Pavia respectively) serving a district of about 400,000 residents and more than one million professionals commuting daily from suburbs and surrounding areas. All three EDs offer psychiatric emergency service 24/7 and provide treatment for a range of psychiatric conditions.

### 2.2. Study Population and Data Collection

**Figure 1:**
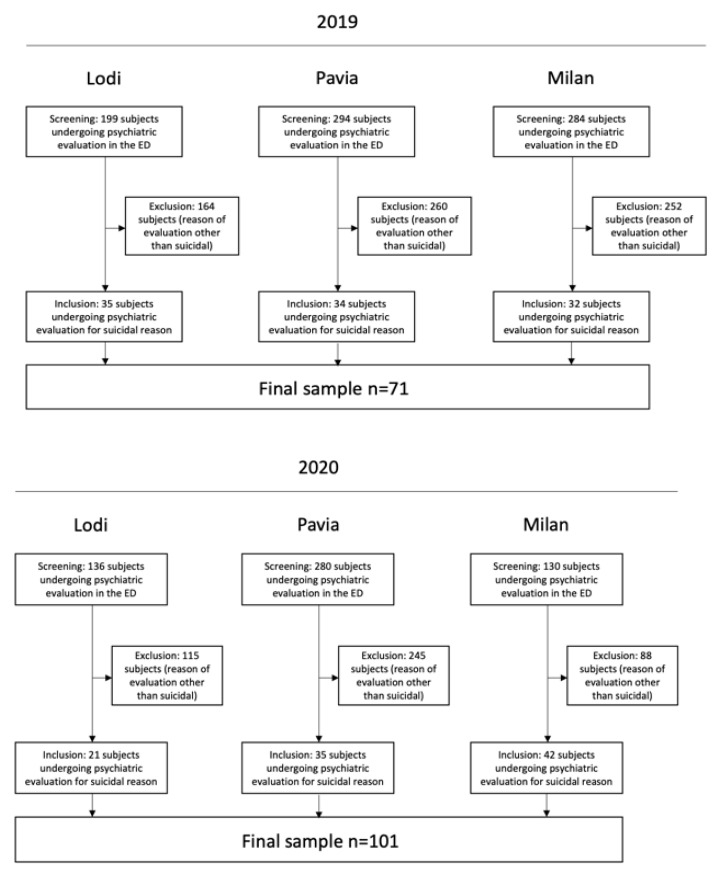
Recruitment Flow-chart.

A retrospective chart review of medical records was carried out at the three EDs using hospitals’ computer databases of emergency rooms reports. All subjects (i) older than 18 years and (ii) accessing the three EDs for suicidality between 8 March and 3 June 2020 were selected for inclusion in the analyses. In addition, subjects meeting the inclusion criteria throughout the same period of 2019 were included as a comparison group. The total number of subjects referring to the EDs and going through a psychiatric evaluation during the two periods was also annotated. The flow-chart illustrating the recruitment process is shown in Figure 1.

Data were extracted anonymously including sex, age, nationality (Italian vs. other), marital, cohabitation and occupational status, usual care provider (private/public Mental Health/Addiction Service), history of alcohol and substance use, phase of access (8 March–4 May vs. 5 May–3 June), type of suicidality (suicidal thoughts, suicide attempt, self-injuring, drug ingestion), presence of triggering conflicts, triage priority level (high vs. low), psychopharmacological treatment prescribed before/during/after ED consultation, discharge diagnosis (anxiety/mood/psychotic/personality disorder/no mental disorder-harmful substance use), and admission to the inpatient psychiatric service. The period between 8 March and 4 May 2020, when the number of COVID-19 cases rose and the lock-down measures were implemented, was designated as the peak epidemic stage (phase 1), while the period between 5 May and 3 June, as the outbreak began to subside and the measures of lock-down were removed, was defined as the late-epidemic stage (phase 2). The study was performed in accordance with the principles of the Declaration of Helsinki regarding medical research in humans and it satisfied local research ethical requirements. In particular, the privacy of research subjects and the confidentiality of their personal information were protected by anonymization of all collected data. As a retrospective, non-interventional, low-risk study, the institutional review boards at each participating site approved the study protocol and the local ethic committee was notified before study initiation.

### 2.3. Statistical Analyses

Demographic and clinical characteristics of patients accessing the EDs for suicidality in 2019 and 2020, respectively, were compared using a *t*-test for continuous variables and Chi-square test for categorical variables. The number of accesses for suicidality out of the total number of ED visits were compared between the two years using Chi-square test. Additional analyses were conducted within each year group to compare subjects based on sex, phase of the outbreak (phase 1/phase 2) and site of enrollment. Chi-square test with Odd Ratios (OR) values and 95% confidence intervals (CI) were used to find significant predictors of admission to the psychiatric inpatient unit only for the year 2020. A *p* value of less than 0.05 was considered statistically significant. All statistical analyses were carried out using SPSS, version 26 (IBM, Armonk, NY, USA) [25].

## 3. Results

### 3.1. Characteristics of Patients Accessing the ED for Suicidality during the First Wave of COVID-19 in 2020

Demographic characteristics of patients referred to the ED for suicidality between March 8th and June 3rd 2020 are displayed in Table 1. Overall, 94 subjects accessed the ED for suicidality (22.3% in Lodi-Codogno, 52.1% in Pavia, 25.5% Milan) with 58.5% accessing in Phase 1 and 41.5% in Phase 2. Most of them (77.7%) were Italian, with no differences in the percentage of foreigners/Italians accessing the ED during Phases 1 and 2. Across the three months, the majority of subjects were unemployed and unmarried. As shown in Table 2, half of the subjects did not usually refer to any mental health/addiction service, and the majority (52.1%) were admitted to the ED after an episode of intentional prescription drug ingestion. Overall, 12.8% accessed the ED for current suicide attempt; the distribution of suicide attempts vs. any other suicidality feature did not significantly differ between phase 1 and phase 2 in the overall sample (9.1% of suicide attempts in phase 1 vs. 17.9 in phase 2; Chi-square = 1.608, *p* = 0.205) nor considering each center separately. At the end of ED consultation, the vast majority, 87.2%, received a mental disorder diagnosis, while the remaining 12.8% were discharged with no psychiatric diagnosis/substance harmful use. Thirty subjects (31.9%) were admitted to the psychiatric inpatient unit. Among a range of possible risk factors (sex, taking antidepressants/anxiolytics/mood stabilizers/antipsychotics, suicide attempt vs. others, having/not having a psychiatric diagnosis, self-referred detrimental impact of COVID-19) only female sex (39.7% vs. 19.4%, OR = 2.7, IC 1.0–7.2) and having a psychiatric diagnosis (36.6% vs. 0%, OR = 0.81, IC 0.72–0.91) were shown to be significant risk for being admitted to the psychiatric inpatient unit. Females were also more likely to present with an episode of intentional prescription drug ingestion (*p* = 0.043), while males were more likely to show acute alcohol/drug intoxication; no differences were found in the prevalence of substance abuse. The majority of males did not usually refer to any mental health/addiction service (63.9%), while the majority of women (58.6%) did (chi = 4.502, *p* = 0.034). A significantly higher percentage of men than women were discharged with antipsychotic (22.2% vs. 6.9%, chi = 4.685, *p* = 0.030) and antidepressant (30.6% vs. 13.8%, chi = 3.870, *p* = 0.49) prescription. At the time of discharge from ED, the majority of females (65.9%) were diagnosed with a psychiatric disorder, while the majority of men (66.7%) were diagnosed with harmful substance use/no psychiatric disorder (4.685, *p* = 0.030).

### 3.2. Comparisons between 2020 and 2019

A total number of 777 subjects were referred to the ED and went through PES evaluation in the three centers between 1st March and 31st May in 2019. Of those subjects, 101 (13.0%) did so for suicidality. In the same period of 2020, 546 patients overall accessed the ED and underwent psychiatric consultation, 94 (17.2%) for suicidality, with a statistically significant difference between the two years (Chi-Square: 4.5386; *p* = 0.03). Considering every single center, the difference was not significant for the center of Lodi (14.6% suicide in 2019 vs. 15.4% in 2020, chi: 0.046; *p* = 0.83), nor for Milan (12.8 suicide in 2019 vs. 15.8 in 2020, chi = 0.639, *p* = 0.42), but was so in Pavia (11.6% suicide in 2019 vs. 19.0% in 2020, chi = 5.934; *p* = 0.02). Comparisons of clinical characteristics of patients accessing in 2019 and 2020, respectively, are presented in Table 2. No differences were found for sex, triage priority level and history of substance abuse between the two years. No differences were found about factors triggering suicidality (conflicts with family members vs. anxiety/exacerbation of psychopathology) in the overall sample, nor considering each center separately. However, the difference was significant considering only Phase 2, with 71.8% of subjects accessing in Phase 2 doing so for anxiety/exacerbation of psychopathology, and 42.9% in the same period of the previous year (Chi = 6.345; *p* = 0.012). No differences were found in the percentage of subjects who were admitted to the psychiatric inpatient unit between 2019 and 2020 in the overall sample, nor considering each center or each period separately. No differences were found in the prevalence of each diagnostic group (psychotic disorders, mood disorders, anxiety disorders, personality disorders) as discharge diagnoses between 2019 and 2020. While dichotomizing discharge diagnoses between psychopathological or no mental disorders/harmful substance use, a difference close to significance was found between 2019 and 2020 with 5% of subjects with no mental disorders in 2019 and 12.8% in 2020 (Chi = 3.737, *p* = 0.050).

As shown in Table 3, a significant difference was found between 2019 and 2020 regarding the percentage of subjects treated with any psychotropic drug at the moment of ED consultation, with a minority of patients (26.7%) who were psychotropic drug-free in 2019 compared to 40.4% in 2020 (Chi 4.108, *p* = 0.043). No differences were found between 2019 and 2020 in the type of treatment used before/prescribed after ED consultation, except for patients accessing in 2019 having greater likelihood of being treated with anxiolytic drugs before ED consultation compared to those accessing in 2020 (33.0% vs. 50.5% Chi = 6.130, *p* = 0.013).

## 4. Discussion

The main aim of the study was to compare the characteristics of patients accessing the ED for suicidality during the first wave of COVID-19 in 2020 with those accessing in the same period of 2019 in three Italian EDs differently affected by the SARS-CoV2 outbreak (Codogno, the first struck by the epidemic wave, Pavia and Milan). First, out of all the people referring to the psychiatric services of the EDs, the proportion of consultations due to suicidality was significantly higher in 2020 that in 2019. This finding is in line with previous data suggesting that massive events may trigger suicidality and contribute to the existing literature about the direct and indirect consequences of the pandemic [12,13,14]. Analyses separately carried out for each center further indicated that the difference in the rate of psychiatric consultations due to suicidality in the two years was actually significant in the center of Pavia but not in the centers of Lodi-Codogno and Milan. The absence of significance for the center of Lodi-Codogno may appear in contrast with early exposure to the COVID-19 of this area. Codogno was indeed the first epicenter of the outbreak in Italy and its population was subjected for an especially long time to severe restrictions, social isolation, and risk of infection. However, there is the possibility that a number of people needing psychiatric emergency consultation during the first epidemic wave was shifted to the nearby Department of Pavia, in which the number of accesses for psychiatric consultation was in fact especially high compared to the other two centers and significantly higher than in 2019. While no differences were found regarding the reasons triggering suicidality in the phase 1, a greater proportion of suicidal behaviors during the phase 2 was caused by relapsing psychopathology—instead of being triggered by interpersonal problems—compared with the same period of 2019. One hypothesis could be that the stress suffered during the epidemic phase contributed to starting the process of relapse that became fully manifested only during the post-epidemic phase [26]. On the other hand, it is also possible that feelings of uncertainty and the fear of contagion withheld people with relapsing symptoms from seeking for help in the ED during the peak epidemic phase, with some sort of rebound in patients with relapsing psychopathology as soon as the contagion started to subside in the post-epidemic phase [26]. This interpretation could also be in line with the decrease in the overall number of psychiatric emergency consultations from 2019 to 2020, confirming that the epidemic wave led to fear and avoidance of the ED to some extent. This also stands as a caveat to the increased suicidality in 2020, as the total number of accesses for suicidality remained substantially constant from 2019 to 2020, while the increase percentage of accesses for suicidality was mostly due to a drop in the amount of psychiatric emergency consultations for other reasons.

The overall severity of suicidal gestures was not more severe in 2020 than in 2019. In fact, no differences between years were found in the percentage of psychiatric emergency visits leading to inpatient treatment admission nor in the distribution of different features of suicidality. Dichotomizing discharge diagnoses between psychopathological or no psychopathological, a difference very close to significance by year was found, with a higher proportion of subjects with no mental disorders accessing for suicidality in 2020 than in 2019. Although not significant, this result suggests the need of further investigation and might indicate a large impact of COVID-19 on psychological wellbeing and suicidal behaviors, severely involving not only people with preexisting psychiatric disorders but also a broader group of people somehow vulnerable to the multifaced effect of the pandemic [27,28]. Such a hypothesis is corroborated by the finding of a greater proportion of subjects free from psychopharmacological treatments accessed in 2020 compared with 2019 and by the greater likelihood of being already treated with anxyolitics among subjects seeking consultation in 2019. Interestingly, a recent study hypothesized a mediating role of HPA activity and inflammation between social isolation and suicidality, providing a possible neurobiological framework to the increased suicidality observed in our study [29].

Some noteworthy features also emerged from cross-sex comparisons within the 2020 year. While in 2019 sex was not shown to affect the probability of being admitted to a psychiatric inpatient unit, in 2020 women were more likely than men to receive inpatient treatment as a result of psychiatric emergency consultation. Moreover, women were more likely to be already in treatment in outpatient mental health or addiction services at the time of consultation and to receive a mental disorder diagnosis at the time of discharge. On the other hand, men were mostly not referring to any community-based service and were more likely to seek for help in the ED after suicidal behavior arising from substance use unrelated to any mental disorder. Overall, females looked especially prone to enact suicidal gestures in the context of a preexisting mental disorder, while men appeared likely to show a suicidal behavior mostly independently from mental illness, highlighting the role of environmental risk factors for suicidality in the context of the COVID-19 pandemic. For example, one hypothesis could be that men are more vulnerable to react with externalizing behaviors to psychological stress and less likely to seek psychological and social support [30]. Additionally, the economic and employment strains following the pandemic could especially affect men as far as they are bound to endorse the traditional role of family breadwinners [31]. Indeed, unemployment has been shown to contribute differently to the risk of suicide among men and women [32,33].

We acknowledge some limitations of this study. First, results would be more reliable if comparisons were made not only with 2019 but with multiple years preceding the pandemic. Second, the sample is relatively small, and data were brought from few EDs in Northern Italy. Despite involving three departments differently hit by the pandemic in Lombardy, results cannot be assumed to be representative of the whole region. Third, the study has a retrospective design and data were not collected for the purpose of research. Further, no distinction about the violent/not violent nature of suicidal behaviors was provided. Lastly, as cases were recruited based on ED records, we could not include data about completed suicide, lacking information about the extreme end of suicidal spectrum both in 2019 and 2020.

## 5. Conclusions

Our study suggests that the proportion of subjects accessing the ED for suicidalty during the first wave of the COVID-19 epidemic was significantly higher in 2020 compared to the same period of 2019. Although this could be due to an overall drop of ED accesses during the first peak epidemic phase, we also found that a greater percentage of subjects enacting suicidal behaviors during this period was psychotropic drug-free compared to 2019, suggesting that suicidality might not be directly related to a pre-existing treated mental disorder. Our study provides some hints to be used by clinicians managing suicidality during the ongoing emergency and may be of primary ground for further studies on suicidality arising during large-scale health emergencies. Further investigations in later phases of the ongoing pandemic will help to elucidate the overall impact of such emergency on suicidal spectrum behaviors.

## Figures and Tables

**Table 1:** Demographic characteristics of the study sample. Values presented in parentheses are per cent, unless otherwise indicated.

	Year 2019	Year 2020	Chi Square	Sig.
	(*n* = 101)	(*n* = 94)		
**Sex**			0.035	0.852
female	61 (60.4)	58 (61.7)		
male	40 (39.6)	36 (38.3)		
**Nationality**			0.157	0.692
Italian	76 (75.2)	73 (77.7)		
Other	25 (24.8)	21 (22.3)		
**Occupation**			3.338	0.503
employed	17 (16.8)	13 (13.8)		
unemployed	40 (39.6)	41 (43.6)		
student	12 (11.9)	6 (6.4)		
retired	10 (9.9)	7 (7.4)		
other/not known	22 (21.8)	27 (28.7)		
**Marital status**			3.955	0.412
Married	15 (14.9)	18 (19.1)		
unmarried	54 (53.5)	48 (51.1)		
separated/divorced	10 (9.9)	14 (14.9)		
widowed	7 (6.9)	7 (7.4)		
other/unknown	15 (14.9)	7 (7.4)		
**Cohabitation status**			9.407	0.052
partner/children	25 (24.8)	40 (42.6)		
parents/siblings	21 (20.8)	20 (21.3)		
alone	24 (23.8)	19 (20.2)		
institution	18 (17.8)	9 (9.6)		
other/unknown	13 (12.9)	6 (6.4)		
**Phase of access**			0.966	0.326
8 March–4 May	66 (65.3)	−58.5		
5 May–3 June	35 (34.7)	−41.5		
			**T**	**Sig.**
**Age** (mean, SD)	42.5 ± 17.6	42.4 ± 15.4	0.051	0.959

**Table 2:** Clinical characteristics of the study sample.

	Year 2019	Year 2020	Chi^2^	Sig.
	(*n* = 101)	(*n* = 94)		
**Usual care provider**			0.305	0.859
None	49 (48.5)	48 (51.1)		
Public/private MHS^+^	40 (39.6)	37 (39.4)		
Addiction Service	12 (11.9)	9 (9.6)		
**History of alcohol substance abuse**	26 (25.7)	27 (28.7)	0.219	0.64
**Triage priority level**			0.005	0.945
high	51 (50.5)	47 (50)		
low	50 (49.5)	47 (50)		
**Conflicts triggering** **suicidality**	47 (46.5)	38 (40.4)	0.739	0.39
Suicidality *				
Suicidal thoughts	16 (16)	19 (20.2)	0.582	0.446
Suicide attempt	1 (1)	3 (3.2)	1.174	0.279
Self-injuring	21 (21)	14 (14.9)	1.222	0.269
Drug ingestion	54 (54)	49 (52.1)	0.068	0.794
**Discharge diagnosis**				
Anxiety disorder	7 (6.9)	6 (6.4)	0.023	0.878
Mood disorder	38 (37.6)	31 (33)	0.459	0.498
Psychotic disorder	2 (2)	7 (7.4)	3.305	0.069
Personality disorder	49 (48.5)	38 (43.7)	1.289	0.256
No mental disorders/harmful substance use	5 (5)	12 (12.8)	3.737	0.05
**Admission to psychiatric inpatient care**	32 (31.7)	30 (31.9)	0.001	0.972

^+^ MHS: Mental Helath Service. * all the features listed relate to the aim of ending own life. ‘Suicide attempt’ refer to a potentially life-threatening behavior; ‘self-injuring’ and ‘drug ingestion’ refer to self-harming acts with a declared suicidal intent but lacking life-threatening potential.

**Table 3:** Treatment characteristics of the study sample.

	Year 2019 (*n* = 101)	Year 2020 (*n* = 94)	Chi^2^	Sig.
**Psychotropic treatment at the moment of ED consultation**				
Any psychotropic treatment	74 (73.3)	56 (59.6)	4.108	0.043
Anxiolytics	51 (50.5)	31 (33)	6.130	0.013
Antidepressants	43 (42.6)	41 (43.6)	0.022	0.883
Antipsychotics	33 (32.7)	23 (24.5)	1.601	0.206
Mood stabilizers	12 (11.9)	9 (9.6)	0.270	0.604
**Psychotropic treatment administered during ED consultation**				
Anxiolytics	12 (11.9)	26 (27.7)	7.725	0.005
Antidepressants	0 (0)	1 (1.1)	1.080	0.299
Antipsychotics	6 (5.9)	5 (5.3)	0.035	0.851
Mood stabilizers	0 (0)	2 (2.1)	2.171	0.141
**Psychotropic treatment prescribed at discharge from PES**				
Anxiolytics	17 (16.8)	15 (16)	0.027	0.869
Antidepressants	22 (21.8)	19 (20.2)	0.072	0.788
Antipsychotics	14 (13.9)	12 (12.8)	0.051	0.822
Mood stabilizers	5 (5)	3 (3.2)	0.383	0.536

## Data Availability

The data presented in this study are available on request from the corresponding author. The data are not publicly available due to their collection from the hospital database. Data are not available in a publicly accessible repository.
